# Psychological processes in the experience of hereditary angioedema in adult patients: an observational study

**DOI:** 10.1186/s13023-020-01643-x

**Published:** 2021-01-09

**Authors:** Livia Savarese, Maria Bova, Assunta Maiello, Angelica Petraroli, Ilaria Mormile, Mauro Cancian, Riccardo Senter, Andrea Zanichelli, Giuseppe Spadaro, Maria Francesca Freda

**Affiliations:** 1 Department of Humanities, University “Federico II”, Naples, Italy; 2 Department of Translational Medical Sciences and Center for Basic and Clinical Immunology Research (CISI), University of Naples Federico II, Naples, Italy; 3 Department of Medicine, University of Padua, Padua, Italy; 4 ASST Fatebenefratelli Sacco, Department of Biomedical and Clinical Sciences, “Luigi Sacco” Hospital, University of Milan, Milan, Italy

**Keywords:** Hereditary angioedema, Psychological processes, Stress, C1 inhibitor, C1 inhibitor deficiency

## Abstract

**Background:**

Hereditary angioedema associated to C1 inhibitor deficiency (C1-INH-HAE) is a pathological condition characterized by episodes of subcutaneous swelling and it is frequently associated with discomfort and social impairment of the patients, due to the anxiety experienced for an unpreventable manifestation of an attack during daily life. In children increased level of stress and alexithymia have been associated to C1-INH-HAE, and the latter correlated also with the severity of the disease. We hypothesized that the involvement of psychological issues may impact on the severity of C1-INH-HAE in adult patients as well, interfering with their ability to engage with the management of the disease.

**Methods:**

28 adult patients with C1-INH-HAE were evaluated for clinical (C1-INH-HAE Severity Score) and psychological factors (alexithymia, emotion regulation, stress, patient health engagement, general severity index) by means of validated questionnaires.

**Results:**

Mean age (standard deviation [SD]) was 45 (11) years and time from diagnosis was 20 (12) years. The mean C1-INH-HAE severity score was 6.4. Alexithymia was absent in 22 (78%) patients. Moderate and high stress levels were present in 17 (61%) and 4 (14%) patients, respectively. Moderate-high discomfort was experienced by 9 (36%) patients and a discomfort beyond the clinical attention threshold was shown by 3 (12%) patients. Stress correlated with patient health engagement and with psychological discomfort.

**Conclusions:**

In C1-INH-HAE, patients health engagement and moderate-high psychological discomfort are linked with stress but not with the severity of the disease or alexithymia. A better patient health engagement may be a target for psychological intervention in clinics to ameliorate the stress perceived by C1-INH-HAE patients.

## Background

Angioedema is a hereditary or acquired condition characterized by episodes of swelling of cutaneous and subcutaneous tissue [1]. Despite several mediators linked to the development of the disease have been identified, bradykinin is the best characterized for some types of hereditary angioedema (HAE) [2]. HAE can be associated to a C1 inhibitor deficiency, which may be quantitative (type I C1-INH-HAE) or functional (type II C1-INH-HAE) [2]. External swelling causes disfiguration, discomfort and social impairment, while swelling involving the abdominal or laryngeal tissues may cause pain and it is potentially life-threatening [2]. Frequency, severity and site of attacks are extremely variable among patients and also during the patient’s life, and identification of triggering events is usually difficult. Patients may experience difficulties in carrying out daily living activities because of frequent attacks or due to anxiety for an unpreventable event [3].

Psychological processes in the experience of HAE could be relevant in the course of the disease because they could trigger attacks, impact on quality of life and interfere with coping; nevertheless, the subject has been scarcely investigated.

Depression seems a frequent issue in patients with HAE: 42% of patients presented with depression in a study by Bygum et al. [4], and high levels of depression were found in 100% of 26 patients with HAE by Fouche et al. [5]. These authors proposed that depression may be linked to angioedema by common neurobiological and inflammatory events.

Recently, emotional stress has been reported by patients as the most common trigger factor for angioedema attacks, and chronic stress seems to modify disease activity too [6–8]. We have previously found that perceived stress was worse in pediatric patients with angioedema compared with pediatric patients with other chronic diseases [9]. In addition, in the same sample of children, perceived stress was correlated with alexithymia, i.e., the difficulties in recognizing and naming one’s own emotions [10]. Alexithymia was correlated with angioedema severity as well.

Recent literature emphasizes the importance of patient health engagement (PHE) on the adjustment to chronic disease. PHE can be described as “the level individuals are engaged in care management according to their emotional, cognitive, and behavioral mindset” [11]. According to Graffigna et al., when individuals receive a serious diagnosis, they might not be able to engage fully in care management because of destabilizing emotional effect on health knowledge. As patients gain knowledge, the positive impact on emotion regulation influences their ability to engage in managing their disease condition [11].

Based on previous findings on children [8, 12], we hypothesized that alexithymia, stress and emotion regulation may have an impact on angioedema severity also in adults. Indeed, perceived stress, alexithymia and emotion regulation may be strictly related processes influencing the severity of the disease. This work reports the results of a study on adult subjects with C1-INH-HAE aimed at evaluating the levels of emotion regulation, alexithymia, perceived stress, psychopathologic symptoms and at investigating the relation of these processes with angioedema severity and with patients’ health engagement. Moreover, the study investigated whether a better health engagement is associated with reduced disease severity and better emotion regulation.

## Patients and methods

### Study design

This was an exploratory, observational study conducted in adult patients with C1-INH-HAE. Patients diagnosed with C1-INH-HAE, aged ≥ 18 years, and followed-up in the Italian Referral Centers of Naples, Padua and Milan were recruited. All procedures performed in this study were in accordance with the ethical standards of the study center and with the 1964 Helsinki Declaration and its later amendments or comparable ethical standards. Prior to study participation, patients signed an informed consent form. The study protocol was approved by the Ethics Committee of the University Hospital Federico II of Naples, Italy (Protocol no. 116/19).

### Assessments

Demographic and clinical data were collected from patient medical charts and diaries.

Assessments were performed during a face-to-face meeting. The different scales were concomitantly evaluated by the patients during the meeting.

Clinical and psychological factors were evaluated using validated Italian translations of the following tools: the C1-INH-HAE Severity Score [13]; the Toronto Alexithymia Scale (TAS) [10]; Emotion Regulation Checklist (ERC) [14]; Perceived Stress Scale (PSS) [15, 16]; Patient Health Engagement Scale (PHE-S) [11], Symptom Checklist-90 R (SCL-90 R) [17].

The Severity Score for C1-INH-HAE is a clinical score (cumulated 0–10 points) evaluating the severity of attacks, disease activity, therapeutic control and impact on quality of life. It was calculated based on the following factors: age at disease onset, occurrence of skin edema, of painful abdominal edema, of laryngeal edemas, other clinical manifestations and long-term prophylaxis [13]. The higher the score resulted, the higher the severity experienced by the patient.

The TAS is a 20-item self-reported questionnaire which evaluates alexithymia, the difficulty in recognizing and naming and describing one’s own emotions. A score of < 51 indicates absence of alexithymia, a score of 52–60 indicates a possible alexithymia, and a score of > 61 indicates alexithymia [10].

The ERC is a 10-item self-reported questionnaire that evaluates processes of emotion regulation; it has two subscales: reappraisal and suppression. The mean values for both males and females within the normative samples can be referred as cut-off values for the index [14].

PSS, a 10-item self-reported scale, measures the degree to which situations in one’s life are appraised as stressful. Stress was assessed as low with a score < 13, moderate with a score of 14–26, and as highly perceived when the score was > 27 [16, 17].

The PHE-S measures the following psychological experiential features of patient’s engagement in one’s health: blackout, arousal, adhesion and eudaimonic project [11]. The score represents the different type of engagement of the patient with the disease, with the first two definitions corresponding to a lower ability of engagement and the other two to a more effective engagement with the disease.

The SCL-90 R is a 90-item self-reported questionnaire investigating the psychopathology spectra. Answers are interpreted according to nine primary symptom dimensions: somatization, obsessive–compulsive disorder, interpersonal sensitivity, depression, anxiety, hostility, phobic anxiety, paranoid ideation and psychoticism, and according to the global severity index (GSI), which is the summary measure of the nine subscales [18, 19]. SCL-90-R scores were converted to standard T-scores (range 30–80) [20]. T-score range from 40 to 60 represents the normal range (as defined by the mean ± SD).

### Statistical analysis

Data were summarized by descriptive analysis. D'Agostino-Pearson test was used to test the normal distribution of all continuous variables. Means and SD were calculated for continuous variables, while absolute values and frequency (percentage) were calculated for categorical variables. Comparison of mean values was performed by ANOVA analysis. Comparison of categorical data was performed with a Chi-squared test. All analyses were performed with IBM SPSS Statistics for Windows, Version 26.0.

## Results

Overall, 28 adult patients with HAE were included; 20 (71%) were females, mean age was 45 ± 11 years and mean time from diagnosis was 20 ± 12 years.

Descriptive analysis data are presented in Table 1. The mean C1-INH-HAE severity score was 6.4, and median 7 (range 3–9). Alexithymia was absent in 22 (78%) patients, possible in 3 (11%) subjects and present in 3 (11%). Mean TAS-20 was 43.3 and median value was 42.

**Table 1:** Mean and median scores of C1-INH-HAE, Toronto Alexithymia Scale (TAS), Emotion Regulation Checklist (ERC), Perceived Stress Scale (PSS), and Patient Health Engagement Scale (PHE-S) in patients with HAE

Total	Mean (SD)	Median	Min	Max
C1-INH-HAE severity score (n = 28)	6.4	7	3	9
TAS-20 (n = 28)	43.3 (12.9)	42	22	76
ERC (n = 27)	4.4 (0.8)	5	1	7
Reappraisal	5 (1.1)			
Suppression	3.3 (1.1)			
PSS (n = 28)	18.2 (7)	17	7	33
PHE (n = 28)	2.9 (0.65)	3	1	4

Results of the ERC were available for 27 patients and showed a total mean score of 4.4 ± 0.8. The mean score for reappraisal was 5.0 ± 1.1, with a slightly higher score in females (5.2 ± 1.1) than in males (4.5 ± 1.3). The mean score for expressive suppression was 3.3 ± 1.1, without sex differences. Our scores do not differ from the scores of the normative sample, thus not signaling a deficit in emotion regulation.

PSS mean score was 18.2 ± 7.0, with a median score = 17, within a range from 7 to 33. Scores indicated low-level stress in 7 (25%) patients, moderate-level stress in 17 (61%) patients, and perceived stress in 4 (14%) subjects.

**Fig. 1:**
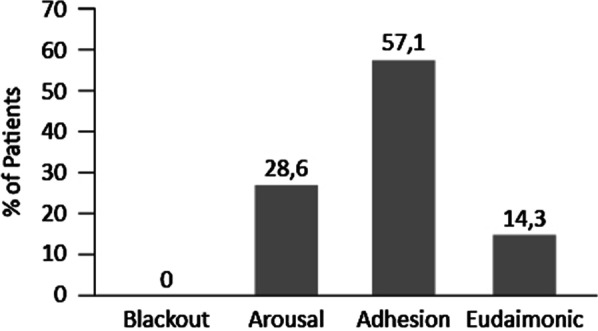
Patient Health Engagement results. Percentage distribution of patients within the different conditions defining Patient Health Engagement

Results of the PHE scale showed that no patient felt as in blackout, 8 (29%) subjects were in an arousal condition, 16 (57%) in adhesion, and 4 (14%) had a Eudaimonic Project (Fig. 1). All patients with PHE equal to “Arousal” (n = 8) perceived at least moderate stress, 75% of patients with PHE equal to “Eudaimonic Project” (n = 3) did not perceive any stress (Table 2). The distribution of PHE results correlates with the presence of stress (*p* = 0.018) but not with other variables.

**Table 2:** Analysis of Patient Health Engagement (PHE) results by stress

PHE result	No stress (N = 7), n (%)	Stress (N = 21), n (%)	Chi-square (*p *value)
Arousal	–	8 (100%)	
Adhesion	4 (25.0%)	12 (75.0%)	0.018
Eudaimonic Project	3 (75.0%)	1 (25.0%)	

Overall, the T-score for the GSI of SCL-90-R, calculated on 25 patients, assessed that 7 (28%) subjects had no general psychiatric discomfort, 6 (24%) had a normal general discomfort, 9 (36%) had from moderate to high general discomfort and 3 (12%) had a discomfort beyond the clinical attention threshold. Incidence of T-scores for the nine dimensions of the questionnaire is reported in Table 3. Among all the SCL-90-R symptom dimensions, psychoticism is the one that shows the highest percentage of patients involved (52%) in the medium to high level. Statistical analysis showed a correlation between stress and moderate-high psychological discomfort (GSI), and between stress and Psychoticism, of the SCL-90-R (*p* = 0.001) whereas no correlation was found between C1-INH-HAE Severity Score and any of the studied factors (Table 4). Equally, the patient’s health engagement was not different by GSI of SCL-90-R, or presence of stress. Finally, alexithymia did not change with the moderate-high psychological discomfort (GSI) of the SCL-90-R (Table 5).

**Table 3:** SCL-90T scores for each dimension and global severity index

Item	T-scores (n = 25), n (%)			
	< 45	45 ≤ T < 55	55 ≤ T < 65	≥ 65
Somatization	5 (20)	10 (40)	4 (16)	6 (24)
Obsessive–compulsive disorder	8 (32)	9 (36)	6 (24)	2 (8)
Interpersonal sensitivity	10 (40)	10 (40)	2 (8)	3 (12)
Depression	9 (36)	7 (28)	6 (24)	3 (12)
Anxiety	7 (28)	9 (36)	5 (20)	4 (16)
Phobic anxiety	8 (32)	11 (42.3)	2 (8)	4 (16)
Hostility	10 (40)	10 (44)	5 (20)	–
Paranoid ideation	8 (32)	8 (40)	7 (28)	2 (8)
Psychoticism	7 (28)	3 (12)	13 (52)	2 (8)
Global Severity Index (GSI)	7 (28)	6 (24)	9 (36)	3 (12)

**Table 4:** Correlations of clinical and psychological factors

	Mean (SD)	ANOVA (*p* value)
Stress by global severity index and psychoticism in the SCL-90 R		
GSI low (n = 13)	14.0 (4.4)	
GSI high (n = 12)	22.8 (6.6)	0.001
Psychoticism low (n = 10)	13.0 (4.2)	
Psychoticism high (n = 15)	21.7 (6.4)	0.001
C1-INH-HAE Severity Score by global severity index (GSI), psychoticism, stress, and patient’s health engagement (PHE) conditions		
GSI low (n = 13)	5.9 (1.9)	
GSI high (n = 12)	6.8 (1.6)	0.252
Psychoticism low (n = 10)	6.1 (2.1)	
Psychoticism high (n = 15)	6.5 (1.6)	0.623
No stress (n = 7)	6.3 (1.9)	
Stress (n = 21)	6.4 (1.9)	0.911
PHE arousal (n = 8)	6.1 (1.5)	
PHE at least adhesion (n = 20)	6.5 (2.0)	0.689

**Table 5:** Correlations of psychological factors

Patient’s health engagement conditions by psychoticism, global severity and stress				
	Arousal, n (%)	Adhesion, n (%)	Eudaimonic project, n (%)	Chi-square *p *value
Psychoticism low (n = 10)	2 (20)	6 (60)	2 (20)	
Psychoticism high (n = 15)	6 (40)	8 (53.3)	1 (6.7)	0.430
GSI low (n = 13)	3 (23.1)	8 (61.5)	2 (15.4)	
GSI high (n = 12)	5 (41.7)	6 (50)	1 (8.3)	0.583
No stress (n = 7)	0	4 (25)	3 (75)	
Stress (n = 21)	8 (100)	12 (75)	1 (25)	**0.018**

Alexithymia by psychoticism and global severity index (GSI)				
	No alexithymia, n (%)	Possible alexithymia, n (%)	Alexithymia, n (%)	Chi-square *p *value
Psychoticism low (n = 10)	10 (100)	0	0	
Psychoticism high (n = 15)	10 (66.7)	2 (13.3)	2 (20)	0.125
GSI low (n = 13)	12 (92.3)	1 (7.7)	0	
GSI high (n = 12)	8 (66.7)	1 (8.3)	3 (25)	0.152

## Discussion

In this study, we performed an analysis on adult subjects with C1-INH-HAE, with the aim of assessing whether emotions, alexithymia and perceived stress could be related with angioedema severity and patients’ health engagement.

The mean C1-INH-HAE severity score resulted to be moderate in this group of patients.

48% of our sample show moderate to high psychopathological discomfort, whereas the rest of the sample shows low or normal levels of general discomfort. Major areas of symptomatology are psychoticism, which is of clinical relevance in 52% of our sample, thus signaling a relevant psychological vunerability in the area of the continuity of the psychological experience of the self and in problems of isolation and retire. Low to moderate levels are shown for sleep problems, somatization, paranoia, anxiety and depression. These results report of a psychopathological vulnerability in our group that requires to be considered from a clinical perspective. Interestingly, both the moderate–high psychological discomfort and the GSI of SCL 90-R correlate with the stress experienced by the patients.

Perceived stress may turn into psychopathological discomfort or it may be perceived more in those subjects with a higher psychopathological discomfort. The two hypotheses are not necessarily independent, rather they may be read in terms of a vicious circle where one leads to the other and so on. Other parameters like PHE and TAS resulted instead independent from the psychological discomfort and the severity of the disease.

Alexithymia rarely occurred in this group of patients; only three subjects had a high TAS score, and emotion regulation strategies do not differ from the ones of the normative healthy sample’s scores [14], thus not confirming the data previously found in children. These results seem to point out that alexithymia and deficits in emotion regulation characterize the experience of children with chronic diseases due to their developmental stage, whereas adults become able to recognize and regulate their own emotions independently from being affected by the disease.

As expected, health engagement of patients with C1-INH-HAE was linked with stress. Patients facing angioedema in an arousal attitude had a high score of perceived stress, while an attitude of Eudaimonic Project was present in those who lived with angioedema without a critical level of perceived stress.

These results could be read in terms of the PHE assumption: individuals might not be able to engage fully in care management because of destabilizing emotional effect due to lack of health awareness. We may therefore hypothesize that a non-critical level of stress results in the ability to managing the disease condition. In turn, a better knowledge of the disease causes less stress to the patients, re-enforcing the positive loop [11]. This observation has important clinical consequences and may help psychologists to identify a clinical objective of their activity. Recognizing patients’ needs of better knowledge of the disease and helping them to get awareness, may reduce patients’ perceived stress and adjoining secondary health impairment, thus leading to a better engagement in managing of the disease. This evidence is of central importance in the patient experience of C1-INH-HAE which is characterized by a high degree of uncertainty and unpredictability of the attacks. Psychologists can work together with physicians to turn this lack of certainty in patient awareness and ability to identify strategies for flexible management of the disease.

On the contrary, severity of C1-INH-HAE was not found to be associated with stress nor with health engagement. This result highlights that psychological attitude toward the disease seems to be more relevant for patients’ wellness than the disease severity itself.

Overall, our results suggest that a better engagement toward the disease may improve the perception of stress of patients facing C1-INH-HAE, but we were unable to demonstrate an improvement of the degree of angioedema severity in patients with a good engagement and a favorable regulation.

Results obtained on the relationship between disease severity and stress in adult patients were comparable with our previous findings in children affected by C1-INH-HAE [9].

We must acknowledge that this study has the limitation of including a limited sample of patients, due to the rarity of the disease. As a consequence, performing a regression analysis would result not appropriate depending on the related covariate number of the model, reducing therefore the relevance of results. We believe, nonetheless, that a description of psychological processes of patients with C1-INH-HAE may provide important clinical hints, and we propose our patient series as a starting point in this field of research. To our knowledge, these results are in fact the first evidence addressing the complex subject of psychological processes in HAE and provide the first suggestion for clinical practice. It seems important that psychologists caring for patients with C1-INH-HAE would attentively evaluate levels of health engagement and of stress to provide targeted interventions aiming to improve patients’ management and adaptation to the disease and to foster doctor-patient’s relationship.

In conclusion, this study explored the psychological processes of patients with C1-INH-HAE, and found that reduced perceived stress is associated with a favorable health engagement; this result suggests a possible therapeutic target for clinical psychologists dealing with this uncommon chronic condition.

## Data Availability

The datasets used and/or analyzed during the current study are available from the corresponding author on reasonable request.

## Abbreviations

HAE: Hereditary angioedema
C1-INH-HAE: Hereditary angioedema associated to C1 inhibitor deficiency
SD: Standard deviation
TAS: Toronto Alexithymia Scale
ERC: Emotion Regulation Checklist
PSS: Perceived Stress Scale
PHE-S: Patient Health Engagement Scale
GSI: Global severity index
SCL-90 R: Symptom Checklist-90 R

## References

[1] Bova M, De Feo G, Parente R, De Pasquale T, Gravante C, Pucci S (2018). Hereditary and acquired angioedema: heterogeneity of pathogenesis and clinical phenotypes. Int Arch Allergy Immunol.

[2] Cicardi M, Zuraw BL (2018). Angioedema due to bradykinin dysregulation. J Allergy Clin Immunol Pract.

[3] Granero-Molina J, Sánchez-Hernández F, Fernández-Sola C, Jiménez-Lasserrotte MDM, Antequera-Raynal LH, Hernández-Padilla JM (2020). The diagnosis of hereditary angioedema: family caregivers' experiences. Clin Nurs Res.

[4] Bygum A, Aygören-Pürsün E, Caballero T (2012). The hereditary angioedema burden of illness study in Europe (HAE-BOIS-Europe): background and methodology. BMC Dermatol.

[5] Fouche AS, Saunders EF, Craig T (2014). Depression and anxiety in patients with hereditary angioedema. Ann Allergy Asthma Immunol.

[6] Zotter Z, Csuka D, Szabo E, Czaller I, Nebenfuhrer Z, Temesszentandrasi G (2014). The influence of trigger factors on hereditary angioedema due to C1-inhibitor deficiency. Orphanet J Rare Dis.

[7] Zotter Z, Nagy Z, Patócs A, Csuka D, Veszeli N, Kőhalmi KV (2017). Glucocorticoid receptor gene polymorphisms in hereditary angioedema with C1-inhibitor deficiency. Orphanet J Rare Dis.

[8] Freda MF, Savarese L, Bova M, Galante A, De Falco R, De Luca PR (2016). Stress and psychological factors in the variable clinical phenotype of hereditary angioedema in children: a pilot study. Pediatr Allergy Immunol Pulmonol.

[9] Savarese L, Bova M, De Falco R (2018). Emotional processes and stress in children affected by hereditary angioedema with C1-inhibitor deficiency: a multicenter, prospective study. Orphanet J Rare Dis.

[10] Taylor GJ, Bagby RM (2004). New trends in alexithymia research. Psychother Psychosom.

[11] Graffigna G, Barello S, Bonanomi A, Lozza E (2015). Measuring patient engagement: development and psychometric properties of the Patient Health Engagement (PHE) Scale. Front Psychol.

[12] Freda MF, Savarese L, Dolce P, De Luca PR (2019). Caregivers’ sensemaking of children’s hereditary angioedema: a semiotic narrative analysis of the sense of grip on the disease. Front Psychol.

[13] Bygum A, Fagerberg CR, Ponard D, Monnier N, Lunardi J, Drouet C (2011). Mutational spectrum and phenotypes in Danish families with hereditary angioedema because of C1 inhibitor deficiency. Allergy.

[14] Balzarotti S, John OP, James Gross JJ (2010). An Italian adaptation of the emotion regulation questionnaire. Eur J Psychol Assess.

[15] Cohen S, Spacapan S, Oskamp S (1988). Williamson G. perceived stress in a probability sample of the United States. The social psychology of health.

[16] Cohen S, Kamarck T, Mermelstein R (1983). A global measure of perceived stress. J Health Soc Behav.

[17] Sarno I, Preti E, Prunas A, Madeddu F. SCL-90-R Symptom Checklist-90-R Adattamento Italiano. Firenze: Giunti, Organizzazioni Speciali (2011). http://hdl.handle.net/10281/19179.

[18] Derogatis LR. SCL-90-R: Administration, scoring and procedures. Manual II for the Revised Version and Other Instruments of the Psychopathology Rating Scale Series (1983). http://www.worldcat.org/oclc/30835138.

[19] Prunas A, Sarno I, Preti E, Madeddu F, Perugini M (2012). Psychometric properties of the Italian version of the SCL-90-R: a study on a large community sample. Eur Psychiatr.

[20] Chinese P, Thorndike R, Salkind NJ (2008). T scores. Encyclopedia of educational psychology, vol 1.

